# NMR Precision Metabolomics: Dynamic Peak Sum Thresholding and Navigators for Highly Standardized and Reproducible Metabolite Profiling of Clinical Urine Samples

**DOI:** 10.3390/metabo14050275

**Published:** 2024-05-10

**Authors:** Alessia Trimigno, Nicole R. Holderman, Chen Dong, Kari D. Boardman, Jifang Zhao, Elizabeth M. O’Day

**Affiliations:** Olaris, Inc., Framingham, MA 01702, USA

**Keywords:** NMR, metabolomics, urinary biomarkers

## Abstract

Metabolomics, especially urine-based studies, offers incredible promise for the discovery and development of clinically impactful biomarkers. However, due to the unique challenges of urine, a highly precise and reproducible workflow for NMR-based urine metabolomics is lacking. Using 1D and 2D non-uniform sampled (NUS) ^1^H-^13^C NMR spectroscopy, we systematically explored how changes in hydration or specific gravity (SG) and pH can impact biomarker discovery. Further, we examined additional sources of error in metabolomics studies and identified Navigator molecules that could monitor for those biases. Adjustment of SG to 1.002–1.02 coupled with a dynamic sum-based peak thresholding eliminates false positives associated with urine hydration and reduces variation in chemical shift. We identified Navigator molecules that can effectively monitor for inconsistencies in sample processing, SG, protein contamination, and pH. The workflow described provides quality assurance and quality control tools to generate high-quality urine metabolomics data, which is the first step in biomarker discovery.

## 1. Introduction

Altered metabolism is linked to nearly every human disease [1,2], making metabolomics ideal to explore the nexus of gene-environment interactions and to discover biomarkers for disease diagnosis, prognosis, and treatment response. Levels of metabolites within human biofluids change continuously in response to genetic and environmental inputs and thus provide the most direct, real-time readout of phenotype [1]. As a result, the field is advancing quickly, with numerous metabolomics studies observing a strong correlation between metabolite levels and specific disease states including for Alzheimer’s disease [3], type 2 diabetes [4], myocardial infarction and stroke risk [5], prostate cancer subtypes [6], early diagnosis of breast [7] and pancreatic cancers [8] and more.

Indeed, a search of PubMed publications over the last 5 years reveals that metabolomics literature is growing at twice the rate of other omic technologies (Figure S1). The flurry of metabolomics research suggests the potential for far-reaching applications, however like any maturing field, there will be hiccups and growth opportunities as best-practices continue to develop. A “reproducibility crisis in metabolomics” is being actively discussed after numerous studies have highlighted limited reproducibility within labs [9], between labs [10], and from one study to the next. For example, a recent meta-data analysis of 24 metabolomics studies in pancreatic cancer revealed disappointing overlap among studies [11], calling into question the clinical utility of results. The root cause underpinning the reproducibility challenges is multi-factorial but includes limited quality control, small sample sizes, with few if any validation cohorts, varied levels of statistical rigor to define hits and both discrepancy and limited reporting on sample handling and preparation.

Metabolites are exquisitely sensitive to genetic, biological, and environmental changes [12,13], which is why they are powerful biomarkers for disease, but it is also what leads to challenges pertaining to reproducible measurements. Sample type, patient cohort, sample collection procedures, sample processing technique, analytical platform (NMR or MS) and statistical analysis (feature selection approach, normalization, and model for prediction) all greatly influence metabolomic study results.

Instead of being discouraged, this is pivotal moment for the field to unite around standardization and reporting so metabolomics can reach its full potential. We have previously demonstrated that non-uniformed sampled (NUS) ^1^H-^13^C HSQC profiling of metabolites provides highly reproducible, semi-quantitative data for a broad range of metabolites in the low micromolar range with a coefficient of variation (CV) of less than 20% [14]. However, while the NMR measurement itself is highly reproducible, there are numerous factors beyond the instrument that can introduce unwanted variations in results. Here, we report a workflow for precision NMR metabolomics in urine-based clinical samples.

Urine is of particular interest for biomarker discovery due to its non-invasive collection, ability to be collected at home, relatively low concentrations of proteins and lipids compared to other biofluids, and comprehensive representation of metabolites arising from numerous molecular pathways in the body [15,16]. However, a unique challenge of urine is that it is not homeostatically regulated. Urine pH can vary from 4.5 to 8 [17,18], and metabolite concentration may vary based on hydration status by as much as 15-fold [19]. These variations can introduce unwanted bias in clinical studies unless addressed. Further, as large sample sizes are often needed to attain the desired statistical power [20], clinical samples are often prepared by different operators, from different laboratories, and across different time points, which can collectively introduce additional sources of undesired and often unknown variation.

The generation of high-quality data is essential for identifying new biomarkers and maximizing the potential of newly emerging computational approaches like artificial intelligence (AI). There is a misconception that AI will be able to unearth new findings by simply gathering more data. The adage “garbage in garbage out” holds true. AI absolutely has the potential to uncover clinically actionable insights, but high-quality data is a pre-requisite. In this study, we present a novel standard operating procedure (SOP) that utilizes dynamic sum-based thresholding for peak picking in combination with “Navigators” or molecules that identify samples in which natural variation, errors, or biases could be affecting data quality. NMR peak picking traditionally involves a relatively arbitrary signal to noise (S/N) cutoff of ~10×. For urine clinical samples with a wide variation in hydration, this can lead to both the inclusion of unwanted noise or artifacts and the exclusion of real metabolite peaks. We show that a dynamic sum-based peak thresholding provides the most robust results independent of hydration. Further, we show that the Navigators can serve as helpful checkpoints for increased quality control and quality assurance prior to biomarker analysis.

## 2. Materials and Methods

### 2.1. Urine Samples Preparation

Urine samples were obtained by Innovative Research (Innovative Research Inc., Novi, MI, USA) and UTAK (UTAK, Valencia, CA, USA). Proteins and macromolecules were removed and metabolites were extracted via methanol/chloroform precipitation. A 7.2 mM solution of Navigator 1 was added to the sample at the beginning of sample preparation to obtain a 1.44 mM final concentration in the NMR sample. The aqueous layer was partially evaporated under reduced pressure and lyophilized overnight. Lyophilized samples were dissolved in sodium phosphate buffer (pH 7.4) prepared in D_2_O with 0.3 mM deuterated sodium 2,2-dimethyl-2-silapentane-5-sulfonate (D, 98% DSS-d_6_) added for chemical shift referencing and with 0.15 mM 1,1-difluoro-1-trimethylsilanyl methylphosphonic acid (DFTMP) where specified. A total of 200 µL of each dissolved urine sample was then transferred to 3 mm NMR tubes and analyzed. Biological replicates (n = 5) were prepared for each condition tested.

Urine specific gravity (SG) was measured using a refractometer (Palm Abbe Digital Refractometer, MISCO, Solon, OH, USA) and adjusted to lower SG by dilution with water or to higher SG by lyophilizing 5 mL aliquots of urine overnight and resuspending them in varying volumes of water to obtain different SG values. For pH experiments, initial urine sample pH was adjusted to the reported values for high and low pH samples through the addition of 14 M NH_4_OH or 12 M HCl before metabolite extraction.

### 2.2. NMR Data Collection and Processing 

Metabolites were analyzed via 1D ^1^H and 2D ^1^H-^13^C HSQC NMR spectroscopy as previously described [21,22] using a Bruker ASCEND solution-state 600 MHz spectrometer equipped with a liquid helium-cooled Prodigy TCI Cryoprobe (H/F, C, N), using a noesypr1d and hsqcetgpsisp2.2 pulse program with non-uniform sampling (NUS), respectively. All experiments were performed at 298 K with an optimized receiver gain (RG), resulting in an RG of 101 for 2D HSQC spectra. For 1D NOESY spectra, 128 scans were recorded, with a relaxation delay of 2 s, a spectral width of 16 ppm, and a time domain (TD) of 32 K, corresponding to an acquisition time of 1.71 s. In HSQC spectra, a 25% NUS schedule compiled using Poisson gap sampling was employed. The spectra were recorded with 72 scans, a relaxation delay of 0.8 s, a spectral width of 16 × 160 ppm, and a TD of 1024 × 256, corresponding to 0.05 × 0.005 s acquisition time.

One-dimensional spectra were processed on Topspin (Bruker Topspin 3.6.4, Bruker BioSpin, Rheinstetten, Germany). The acquired NUS 2D spectral data was processed and reconstructed using iterative soft thresholding, zero-filled, Fourier-transformed and automatically phase-corrected to yield a final digital resolution of 2048 (N2) × 2048 (N1) points using the NMRPipe software package (Version 11.5) [23]. The processed 2D data was then used to generate peak lists. Metabolite resonances were dynamically binned into clusters using Density-Based Spatial Clustering of Applications with Noise (DBSCAN) [24]. DBSCAN is an unsupervised learning algorithm that partitions data into clusters based on their distance to other points. It identifies closely packed points as clusters and marks outliers in low-density regions. Compared to other clustering methods, it effectively identifies and removes noise, making it useful for data cleaning and outlier detection [24].

### 2.3. Statistical Analysis

Normality of the data was assessed using the Kolmogorov–Smirnov (KS) test. Recognizing the non-normal distribution of metabolite resonances, Kruskal-Wallis (KW) non-parametric one-way analysis of variance (ANOVA) was used to test for significant differences in measured NMR resonances between groups of interest. The test of significance was determined by a *p*-value cutoff (*p* < 0.05) and adjusted based on false discovery rate (FDR) using the Benjamini–Hochberg procedure with a cutoff of 0.05 for multiple hypothesis testing correction. Fold change (FC) was calculated as the ratio of the median intensities of the two groups. A FC cutoff of 1.5 was used to determine significant changes, indicating that metabolites with FC greater than 1.5 or less than 0.67 were considered elevated or diminished, respectively. All statistical analyses were performed using R 3.6.1 with ggplot2 for result visualization [25].

## 3. Results

### 3.1. Influence of Specific Gravity

**Figure 1:**
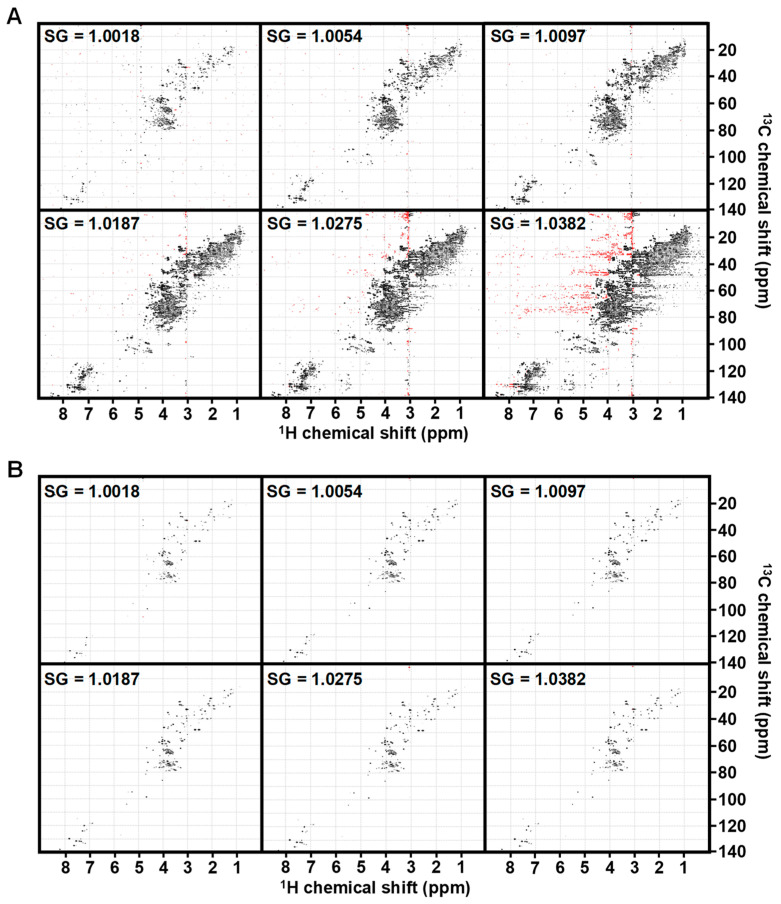
HSQC spectra of identical urine across an SG range adjusted to noise or sum-based cutoff. (**A**) Spectra from a pooled urine sample at 10× noise cutoff across a range of SG from most dilute (1.0018) to concentrated (1.0382). (**B**) The same spectra are plotted using a sum-based threshold of 7 × 10^−4^*sum of positive peak intensity. Spectral colors indicate positive peaks (black) and negative peaks (red).

Urine specific gravity (SG) is a measurement of the density (mass per volume) of a urine sample in comparison with distilled water and is used as an indirect measurement of hydration [26]. To test the influence of SG on false-positive (FP) biomarker hits in urine analysis, the same pooled commercial urine with an SG = 1.0097 was diluted or concentrated to create the 30-fold range of SG typically observed in clinical urine samples (dilutions: 5× and 2×; concentrations: 2×, 4×, and 6×). Metabolites were extracted and analyzed via 1D and 2D NMR. At the routinely applied 10× noise cutoff, the influence of SG is dramatically apparent in the ^1^H-^13^C HSQC spectra, wherein the lower SG samples have fewer peaks and the higher SG samples have many more peaks and noticeable streaking (Figure 1A). Despite all samples originating from the same source urine, after creatinine normalization a KW test of significance with FDR correction identified dozens to even hundreds of statistically different (FDR corrected *p*-value < 0.05 and fold change (FC) > 1.5) metabolite resonances across the different SGs for the same sample (Table 1). This suggests SG could be a major source of FP and that additional steps beyond standard creatinine normalization are warranted.

To see if the standard S/N cutoff was a major contributor to FPs, we manually adjusted the S/N of each SG spectra, so that a comparable number of peaks were observed for those diluted through concentrated samples. In an attempt to link this to an objective spectral characteristic, we measured and calculated 28 variables (Table S1) including peak shape metrics, known metabolite intensities, and ratios between variables. We used a Pearson correlation test to find the variable most correlated with the manual cutoff intensity and calculated the mean ratio of the manual cutoff intensity to this variable across samples. We observed that 0.07% of the total sum of all positive peaks was highly correlated with the manual cutoff, and setting the HSQC plots to this same total sum threshold provided the most consistent data across the SG limits (Figure 1B). Indeed, when we replaced the standard S/N cutoff with a total sum threshold followed by creatinine-based normalization and repeated the FDR-corrected KW test of significance, nearly all of the FP were eliminated (Table 1). These results suggest that dynamic sum-based thresholding can be used to mitigate many FPs associated with SG, especially for SG samples below 1.0275.

**Figure 2:**
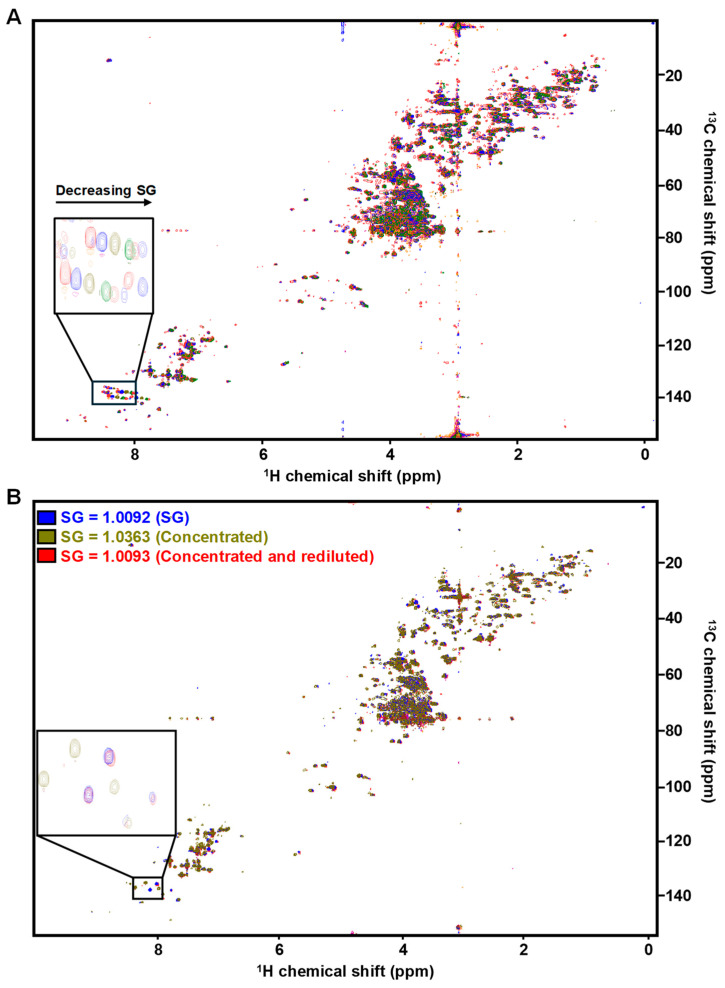
SG impacts chemical shift. (**A**) HSQC spectral overlay of same pooled urine at six different SG values as described in Figure 1. Many resonances display chemical shift perturbation as a function of SG, such as those for histidine and 3-methylhistamine (inset). (**B**) HSQC overlay of the same pooled urine (SG = 1.0092) (blue), concentrated 5× (SG = 1.0363) (green) or concentrated and then re-diluted (SG = 1.0093) (red). Re-diluted samples resolve the chemical shift perturbations observed in concentrated samples.

In addition to metabolite intensity, SG can also affect chemical shift [17]. Dissolved amino acids, salts, and organic acids can undergo proton exchange and metal complexation with endogenous ions such as N^+^, K^+^, Ca^2+^ and Mg^2+^ causing line broadening and/or signal shifting that varies substantially from the free-solution chemical shift of the metabolite [27,28]. We observed chemical shift perturbation as a function of SG for several metabolites including histidine and 3-methylhistamine (Figure 2A). The shifting was more dramatic in the higher SG samples. These perturbations can make clustering or binning features across samples extremely challenging, especially in complex patient samples. We therefore assessed how both FP and chemical shift were affected by performing an SG adjustment, wherein concentrated samples were re-diluted to have an SG similar to the original sample (~1.01). In this case, we observed that again FP were eliminated (Table S2), as were the majority of the chemical shift perturbations (Figure 2B). Having profiled thousands of clinical samples, we believe the SG adjustment step is absolutely critical and provides the most robust results when coupled with dynamic sum-based thresholding.

### 3.2. Navigating Sources of Error in Sample Processing

**Figure 3:**
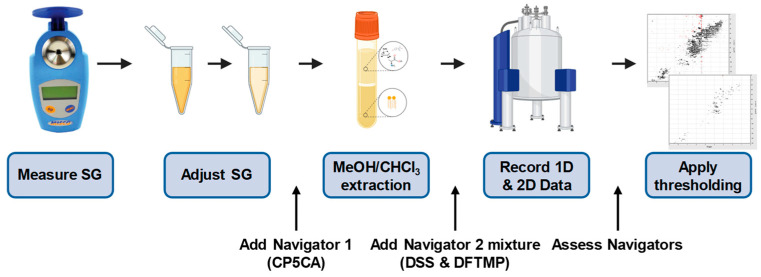
SOP for NMR precision urine metabolomics. SG is measured and adjusted to the range of 1.01–1.02 with ultrapure H_2_O in concentrated samples (SG > 1.02). If SG < 1.0024, the sample is unsuitable for analysis. Navigator 1 is added, and samples are extracted with a MeOH/CHCl_3_ extraction. Navigator 2 mixture, containing DSS and DFTMP, is added and 1D ^1^H and 2D ^1^H-^13^C-HSQC spectra are collected. Spectra are processed in NMRPipe and sum-based thresholding is applied to produce the final spectra for peak picking. Navigator values are evaluated to assess quality of the data.

Many urinary metabolomics studies perform little to no sample processing. We have already described the importance of SG adjustment prior to analysis. In addition, we suggest a modified Folch [29] (methanol/chloroform) extraction to precipitate macromolecules such as proteins and lipids. Proteinuria is a hallmark of kidney dysfunction and is a powerful biomarker itself [30]. However, protein in the urine can negatively affect the relaxation rate of many metabolites and confound analysis [31]. As such, we follow the SOP outlined in Figure 3. The SG of a sample is measured and adjusted as necessary, polar metabolites are extracted into the aqueous phase via methanol/chloroform precipitation of proteins and lipids, the aqueous layer is dried under vacuum pressure and lyophilization before resuspension in a strong phosphate buffer prepared in D_2_O. This method enables 6x concentration of metabolites, which is critical for the ability to perform ^1^H-^13^C HSQC spectroscopy relying on 1.1% ^13^C natural abundance. While we have previously demonstrated this workflow leads to highly reproducible and repeatable results [14], any multi-step process can lead to unwanted variation.

As a result, we set out to identify a molecule that could serve as a Navigator (Nav1) to monitor for potential sources of error in sample processing. The criteria for Nav1 necessitated an inexpensive, non-reactive, non-endogenous metabolite nor known urinary compound, with a limited number of protons and C-H bonds, and a chemical shift of the ^1^H and C-H bond(s) in a spectral region distinct from normal metabolites. After testing several compounds, we selected 2-chloropyrimidine-5-carboxylic acid (CP5CA) as Nav1, a molecule previously used to aid first-order phase correction [32] (Figure S2A). The reference spectrum has ^1^H-^13^C peaks at 8.76 × 161.8 and 9.04 × 163.4 ppm, which is beyond the standard metabolic range. Using a sweep width (SW) of 16 × 160 ppm in ^1^H-^13^C HSQC, the Nav1 reference peaks are folded at 8.76 × 2.06 and 9.04 × 3.5 ppm, respectively, and in the ^1^H spectra the peak at 9.04 is isolated (Figure S2B,C).

**Figure 4:**
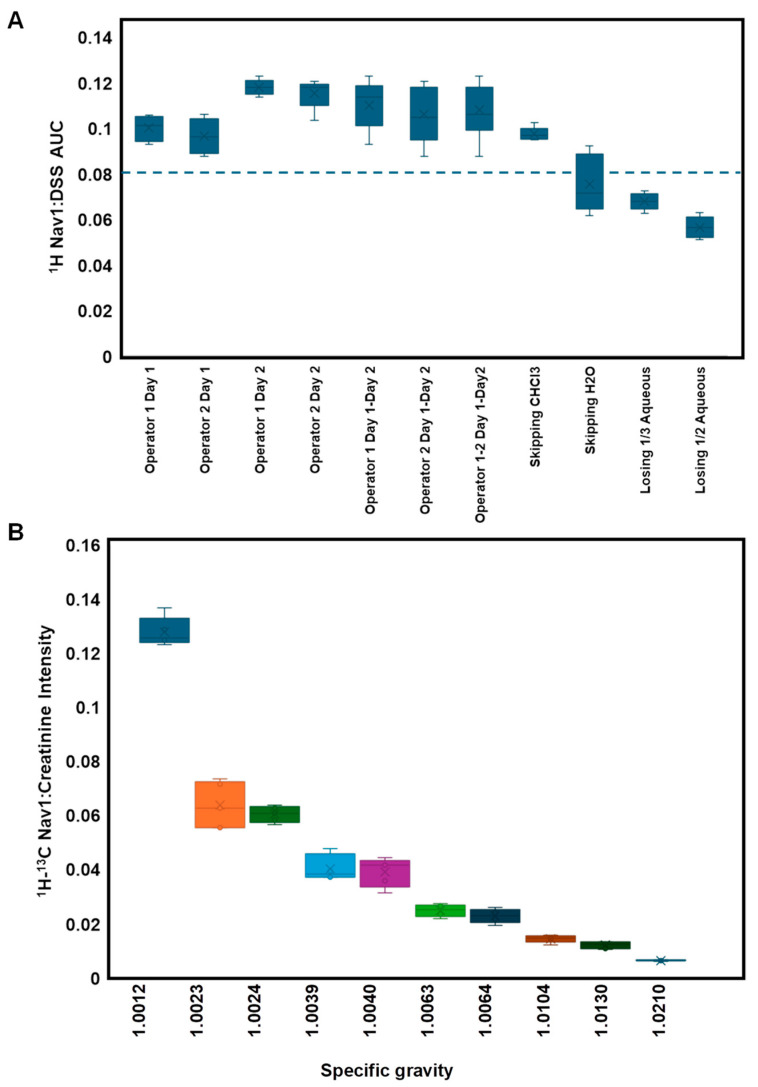
Navigator 1 monitors sample processing biases. (**A**) ^1^H Nav1:DSS AUC ratio is a sensitive indicator for variations in sample processing, wherein a ratio of <0.08 (dotted line) signals suboptimal metabolite extraction. (**B**) ^1^H-^13^C HSQC Nav1:creatinine intensity ratio correlates with SG. Five replicates for each condition were tested with standard deviation represented by error bars.

To demonstrate its efficacy as a Navigator for sources of error, Nav1 was added at the beginning of sample processing and DSS was added in the final step under various conditions (different operators, different days, and altering certain steps in the workflow) (Table S3). The AUC for the Nav1 peak at 9.04 and DSS referenced to 0.0 was calculated for each condition. We observed the AUC of DSS was relatively consistent for all conditions (Figure S3A). The Nav1 AUC and the Nav1:DSS AUC ratio was more sensitive to sample preparation conditions (Figure 4A, Table S3 and Figure S3B). For example, the Nav1 AUC was consistent for different operators on day 1, and while albeit small, significantly higher on day 2. Upon investigation, we determined this was due to a minor inconsistency in preparing Nav1 stock solution on day 2. Despite this subtle change, the Nav1:DSS ratio is highly consistent with a coefficient of variation (%CV) of ~10% across operators and days when the SOP was followed. However, we observed that the Nav1:DSS ratio significantly dropped when processing steps were intentionally altered or omitted. This suggests the Nav1:DSS AUC ratio can be used to flag potential sources of error in sample processing.

We also compared how the intensity of the ^1^H-^13^C Nav1 peak at 9.04 × 3.5 varied with experimental conditions. In particular, we observed a strong correlation between the ratio of Nav1 to creatinine intensity and SG within the range of 1.0023 to 1.0210 (Figure 4B). This relationship broke in very dilute samples (SG = 1.0012), wherein we also noted high levels of noise in the spectra at the sum-threshold (Figure S4). We used this and the results from SG FP analysis to therefore set a range of acceptable SGs for clinical urine samples from 1.002 to 1.02. Using the Nav1:creatinine intensity ratio, we created a linear model to predict SG and were able to accurately predict the SG of 10 blinded samples (Figure S5). This model provides an additional QC checkpoint ensuring that calculated SG is similar to that measured in the lab.

Finally, we tested the stability of Nav1, demonstrating it remains stable at 4 °C for over 30 days (Figure S6). Further, we demonstrated the addition of Nav1 had no change on the intensity of metabolite resonances in the 1D or 2D spectra (Figure S7). Collectively, these results suggest that Nav1, and in particular the ^1^H Nav1:DSS AUC ratio and the ^1^H-^13^C Nav1:creatinine intensity ratio, can be used as Navigators to monitor the quality of sample processing.

### 3.3. Navigating Protein Contamination

Metabolites naturally bind to proteins and/or lipids to initiate catalysis or as allosteric regulators to drive the chemical processes required to support life. These interactions must be disrupted to prevent metabolic breakdown and to provide high-quality measurements of metabolites. The SOP described eliminates the majority of macromolecules, however it is not absolute, and if the protocol is not followed correctly, there is a potential for protein or lipid contamination to persist. Thus, we set out to identify a Navigator molecule to monitor for the presence of protein.

**Figure 5:**
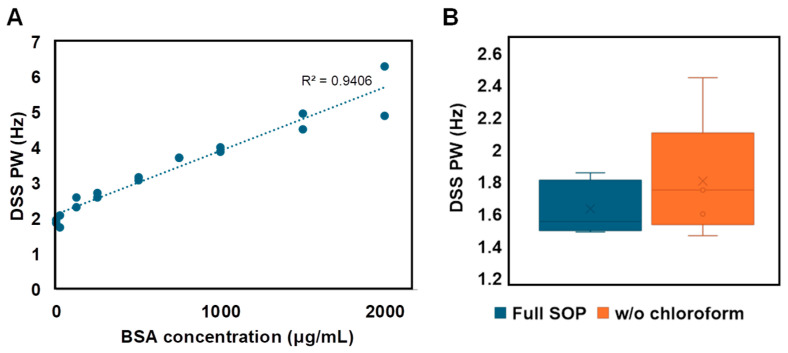
DSS is a Navigator for protein contamination. (**A**) The ^1^H DSS peak width (PW) at half-height increases as a function of protein concentration. (**B**) In metabolites extracted from urine, DSS PW at half-height is ≤1.8 Hz following the full SOP. Samples in which protein precipitation is skipped (i.e., without the chloroform addition step) have a greater range and higher average peak width value. Five replicates for each condition were tested with standard deviation represented by error bars.

We sought to take advantage of the tendency of DSS to interact with molecules to determinate its utility as a Navigator for the presence of protein. We compared the ^1^H half peak line-width of DSS in the presence of increasing amounts of protein (bovine serum albumin, BSA) and observed a strong linear correlation (R^2^ = 0.94), suggesting DSS line-width could be used to monitor for protein contamination (Figure 5A). Indeed, when we failed to precipitate macromolecules by skipping the chloroform step in the SOP, the DSS line width increased significantly, consistent with the presence of protein (Figure 5B). DSS has the added benefit of revealing shimming quality through visualization of peak symmetry and line width. A symmetrical peak with a width of <2 Hz at half maxima should be achieved on the DSS peak for proper shimming. Collectively, these results indicate that DSS line-width can serve as a Navigator for protein contamination and for quality shimming during data acquisition.

### 3.4. Navigating Urine pH

Urine pH typically ranges from 4.5 to 8 [18]. To assess the influence initial urine pH has on metabolite levels, we prepared five replicates each of the same commercial urine and adjusted the pH to 4.99, 6.08 and 8.08. A KW test of significance indicated that of the >160 observed features, none were significantly different between pH groups using an FDR-adjusted *p*-value cutoff of *p* < 0.05 and FC > 1.5 (Table 2). Of note, several features were statistically different using a simple *p*-value cutoff of <0.05, which highlights the benefits of more rigorous cutoffs to prevent FP.

**Figure 6:**
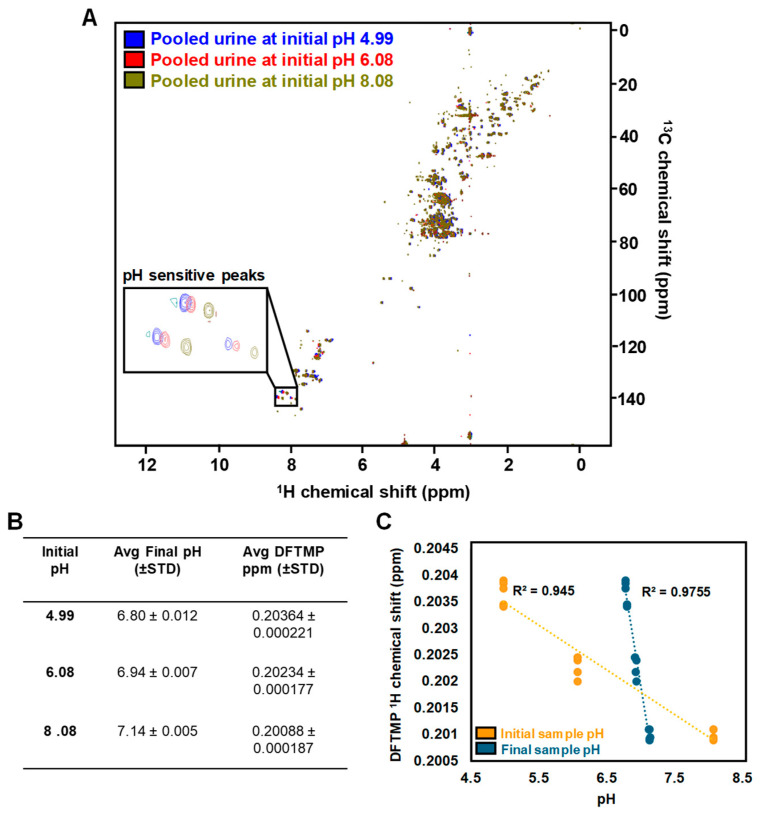
DFTMP is a Navigator for pH. (**A**) ^1^H-^13^C HSQC of pooled urine at pH = 4.99 (blue), 6.08 (red), and 8.08 (green). Inset features an example of pH sensitive peaks (histidine and 3-methylhistamine). (**B**) Initial urine pH led to small but significant changes in the final pH of NMR samples which also caused chemical shift perturbations in DFTMP ^1^H peak position. (**C**) The ^1^H chemical shift of DFTMP shows a strong, inverse relationship to initial sample pH (orange) and final sample pH (blue).

While we were encouraged that initial pH did not lead to FP using our SOP, we did observe chemical shift perturbations associated with initial pH (Figure 6A). Despite all samples being resuspended in the same strong buffer (0.5 M NaPi pH 7.4), initial sample pH caused small albeit significant changes in final pH (Figure 6B). Even these small changes are known to influence the chemical shift of several metabolites, and without knowledge of the final pH of a sample, it can be difficult to cluster features across samples.

Adjusting pH is not amenable to high-throughput studies and direct measurement of the final NMR sample is also not practical. Thus, we set out to identify a molecule that could serve as a Navigator for sample pH. Previous work by Reily et al. demonstrated the chemical shift of 1,1-difluoro-1-trimethylsilanyl methylphosphonic acid (DFTMP) is proportional to pH in serum, plasma, and urine between pH 4.3 and 8.2 [33]. We added 0.15 mM DFTMP with 0.3 mM DSS as part of the final NMR buffer (Navigator 2 mixture) and confirmed its chemical shift is highly correlated with both initial urine pH and final sample pH (Figure 6C). We built a linear model using DFTMP intensity and using histidine as an example of a pH sensitive metabolite, we demonstrated that the DFTMP linear equation could be used to guide the appropriate clustering of histidine across samples (N = 30) with different pH (Figures S8 and S9). These results suggest that DFTMP can serve as a Navigator for pH to empower reliable feature clustering across samples.

### 3.5. Using myOLARIS Navigators for Biomarker Discovery

**Figure 7:**
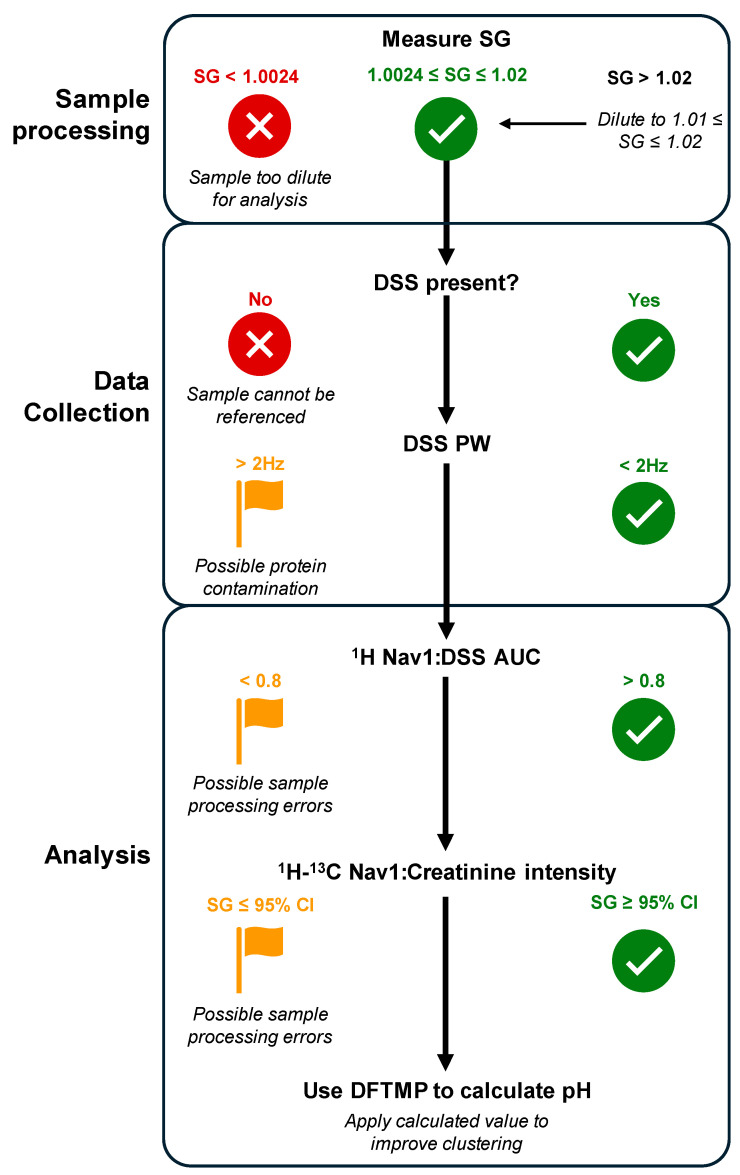
Flowchart of Navigator checkpoints. Sample processing begins with a measurement of SG. Samples with an SG < 1.0024 are too dilute for analysis; if between 1.0024 and 1.02, the sample is ready for preparation, and if SG > 1.02, sample should be diluted to between 1.01 and 1.02. After ^1^H data is collected, observe whether DSS is present. If absent, sample is unable to be referenced and results will be unreliable. If DSS is present, verify the PW is less than 2 Hz. If not, check shim values and flag the sample for possible protein contamination. To analyze data, verify the ^1^H Nav1:DSS AUC ratio is greater than 0.8, otherwise flag the sample for possible errors. For the beginning phase of sample analysis, verify the ^1^H-^13^C Nav1:creatinine intensity ratio estimates SG within the 95% CI of measured SG. Finally, use ^1^H DFTMP chemical shift position to determine initial and final pH of the sample to inform proper clustering. Abbreviations: SG: specific gravity; DSS: deuterated sodium 2,2-dimethyl-2-silapentane-5-sulfonate; PW: peak width at 1/2 height; Nav1: Navigator 1; CI: Confidence interval; DFTMP: 1,1-difluoro-1-trimethylsilanyl methylphosphonic acid.

We present several Navigators to aid in the reliable and reproducible measurement of metabolite from urinary clinical samples. (Table 3). A typical biomarker study will include ~100 urine samples from patients with various phenotypes. Each day, a single operator can prepare ~50 samples according to our SOP, which necessitates splitting the samples into two batches. On day 1, batch 1 samples are thawed, the SG is measured and adjusted as necessary, Nav1 is added and the protocol followed. On day 2, batch 2 of samples are processed in a similar manner. In parallel, on day 2, batch 1 samples are resuspended in NMR buffer containing DSS and DFTMP and added to the NMR for data acquisition. The next day, batch 2 samples are put on the NMR following a similar approach. After data acquisition, the following parameters are evaluated as described in the flowchart in Figure 7. This process ensures only the highest quality data serves as an input for biomarker discovery.

## 4. Discussion

Metabolomics has immense promise to uncover clinically relevant biomarkers. A growing number of mainstream biomedical applications have or are being developed including newborn screening, prediction of cardiovascular disease risk, cancer detection and therapeutics [34]. Due to the unique ability of metabolites to provide a comprehensive and real-time readout of both the genome and environment, this list will continue to expand, especially in personalized medicine applications.

Here, we have described the use of dynamic sum-based thresholding and the use of unique molecules that serve as Navigators to provide highly standardized and reproducible NMR metabolite profiling of clinical urine samples. Unlike other biological fluids such as peripheral blood or cerebral spinal fluid that are homeostatically regulated, urine volume and solute concentration can vary greatly according to hormonal, physiological, dietary and behavioral factors [35,36]. A number of normalization methods have been explored for urine analysis that can be categorized as post-acquisition curative (creatinine, median, probabilistic quotient normalization (PQN), etc.) or pre-acquisition preventative methods (specific gravity, osmolarity), each with different advantages and limitations [36]. Perhaps the most common urine normalization is post-acquisition to creatinine; however, creatinine is shown to greatly vary according to muscle mass, physical activity and renal impairment [36]. In our studies, we demonstrated that using standard S/N cutoff for peak list generation and creatinine normalization is insufficient to mitigate FP associated with varying SG. Rather, we demonstrated that using dynamic sum-based thresholding followed by creatinine normalization yielded the most consistent results. These results are similar to MS studies (which have demonstrated that normalization based on a sum of a subset of signals known as MS Total Useful Signal (MSTUS) or total useful peak area (TUPA) provide highly robust results for urinary analysis [36,37]. A potential limitation of sum-based thresholding that is unique to unbiased NMR profiling like that described here, is that any metabolite resonance can contribute to the sum and thus the cutoff can be influenced by a few very intense peaks, for example high glucose which can occur in diabetic patients or from specific drugs. To mitigate this, careful review of other quality metrics is required including monitoring Navigators and reviewing the number of peaks per spectra, as well as mean, median, min and max intensity. In this manner, it is possible to flag any samples which could require further review/adjustments before proceeding to biomarker discovery.

However, while the post-acquisition sum-based normalization aided in eliminating FP, there were still challenges in interpreting the spectra due to SG-induced chemical shift perturbations. We therefore investigated the utility of a pre-acquisition preventative normalization method where samples above a specific SG value were diluted to be within an acceptable range. We observed this adjustment step coupled with the dynamic sum-based cutoff completely eliminated FP and also prevented chemical shift perturbations associated with SG. Our results are consistent with other studies that show sample SG adjustment can improve the recovery of real sample information and avoid SG-related biases [38]. Our analyses determined adjusting all samples to the exact same SG is not required; rather, it is sufficient to adjust samples within a specific range (1.002 to 1.02). This does prohibit the use of dilute samples (SG < 1.002), which can occur in large cohort studies. However, we believe this quality control serves to set a threshold for the most reliable data. Further, although SG adjustment adds a few additional steps in the lab, previous reports have suggested it can be automated [39].

There can be several unwanted sources of variation during sample processing or data acquisition that are often difficult to detect. Here, we describe the use of Navigator molecules to flag potential issues. For example, the practice of adding internal standards to correct for analyte losses during sample processing and data acquisition is routine in many LC-MS studies [40]. The internal standards must be structurally similar to the analytes of interest, and for complex mixtures like those urine-based metabolomics studies, it can be challenging to find appropriate representative standards for all analytes of interest. While NMR measurements do not necessitate the same type of signal correction, we demonstrate by adding Nav1 at the beginning of sample processing that the Nav1:DSS ratio can detect inconsistencies between batches or operators.

We also show that Nav1:creatinine is highly correlated with SG. This is helpful to provide an additional quality check to ensure the measured and calculated SG are consistent. Of note, extremely high urinary glucose, protein or intravenous contrast dyes can artificially inflate specific gravity [41]. For example, in cases of severe renal disorders, such as uncontrolled diabetes mellitus, every 10 mg glucose/liter could increase SG by 0.004, causing values of up to 1.045–1.05 [42]. Other pathologies, such as diabetes insipidus in which the kidneys cannot properly concentrate urine, could result in lower SG values [42]. Thus, if Nav1:DSS suggests quality sample processing, deviations between the measured laboratory SG and the calculated Nav1:creatinine ratio can serve as a monitor for these types of indications and confounders.

DSS is the most widely accepted internal standard for NMR studies. However, previous reports have demonstrated it has a propensity to interact with biological molecules through electrostatic and hydrophobic interactions [43]. This has led some to suggest that the compound dimethyl-4-silapentane-1-ammonium trifluoroacetate (DSA), which is less likely to bind to proteins, should be used as an alternative reference [44]. However, DSA is more expensive than DSS, and recently both DSS and DSA were shown to interact with micelles [43]. Here, we exploited the natural tendency of DSS to bind proteins to demonstrate that the peak width of DSS can be used to monitor samples for protein contamination.

The influence of pH on the chemical shift perturbation of many metabolites is well-documented [28,45,46]. The nature of this perturbation is complex as not all metabolites nor even all protons in the same metabolite are affected by pH in a similar way. In large clinical studies, this makes it hard to catalogue the same peak as belonging to the same molecule across multiple samples for statistical comparison. For example, the variation in α, β, and δ protons in histidine are shown to vary by 0.04, 0.005, and 0.01 ppm, respectively with pH differences from 6 to 8 [45]. Within the same pH range, the α and β protons of adenosine diphosphate vary by 0.05 ppm [45]. This trend was demonstrated across a majority of amino acids and nucleic acid analogs. Our approach of using 2D NUS ^1^H-^13^C HSQC resolves some chemical shift variability due to the lower sensitivity of ^13^C chemical shifts to sample conditions. However, we still observed large inter-sample peak position variation based on pH. Here, we report the use of DFTMP as a Navigator to determine sample pH, which can empower more robust and reliable clustering of features across samples when coupled with both public and internal libraries of metabolites known to vary across pH. By being able to report pH for each sample, this has the added benefit of enabling urinary pH comparisons across patient cohorts. Food and exercise can influence urine pH, as can different disease states [47,48,49]. For example, high pH in urine may occur in kidney failure and urinary tract infections, while low urine pH can be caused by acidic drugs, diabetes mellitus, chronic nephritis, gout, leukemia and vitamin D deficiency [50]. Thus, DFTMP allows flagging samples with altered pH to enable appropriate comparisons of pH-sensitive metabolite levels across samples but also preserves/reports the pH differences across patients which can inform important biological information.

SOPs are common in industry and clinical settings, but less so in more traditional research laboratories. Due to the unique sensitivity of metabolites, we suggest the field adopt this type of rigorous standardization and documentation more broadly. This will not only enable a higher level of reproducibility but also allow the aggregation of data. Genomics offers a useful example, where both data reporting and repositories have been standardized, bolstering the discovery and development of numerous applications. We are encouraged by the reliability and robustness the current SOP offers for urine clinical biomarker discovery. However, there are several limitations to the study. For example, factors beyond SG, sample processing, protein contamination and pH can impact metabolite chemical shift and intensity such as temperature, presence and variation of divalent cations, sample composition, buffer choice, and many others. The impact of these variables and the ability to uncover Navigator molecules is an area of active research. Further, additional normalization, alignment, and clustering techniques can be assessed. Nonetheless, the protocol and results described provide a strong benchmark to compare future iterations.

## 5. Conclusions

Altered metabolites already provide powerful clinical biomarkers to diagnose disease and guide treatments, with metabolite levels being the most widely assessed indicators of patient health. Among the most common are the measurement of blood glucose for diabetes monitoring, cholesterol for cardiovascular health, blood urea nitrogen (BUN) and creatinine for renal diagnostics, and metabolite panels for inborn errors of metabolism in infants [51]. These assays typically measure only a limited number of metabolites. Translating recent, larger metabolomic studies for use in clinical practice as in vitro diagnostics (IVDs) requires the adaptation of hypothesis-driven metabolomic research to stringent analytical and clinical validation guidelines set by regulatory bodies such as the Food and Drug Administration (FDA) or by the Clinical Laboratory Improvement Amendments (CLIA) which traditionally oversee laboratory developed tests (LDTs) [52]. LipoScience’s LipoProfile Test is an FDA-approved IVD that employs NMR to measure low-density lipoproteins [2], and we at Olaris are on track to launch an NMR-based metabolomic LDT for kidney transplant patients. Other companies such as Metabolon, Numares, Nightingale, and Bruker are also working to translate current “research-use only” IVDs into clinical products [2]. This list will only expand, as advancements in NMR and MS technology, coupled with advanced data analysis tools, have the potential to usher in a new era of clinical insights driving diagnostic innovation. However, reproducibility concerns must be addressed. Here, we demonstrate that using dynamic sum-based peak thresholding and Navigators enables highly standardized and reproducible NMR metabolite profiling of urine samples.

Similar lessons can be applied to study precision NMR metabolomics of other biofluids that may even be less complicated than urine. For example, we have applied the use of Nav1 as a sample prep Navigator and of DSS as a protein indicator in plasma samples (Figure S10). The same principles are applicable wherein deviations from expected ranges can flag samples, however the parameters need to be adjusted for the specific biofluid, wherein for urine a Nav1:DSS of >0.8 indicated sample prep errors; for plasma that value was 0.1. There are also likely biofluid-specific Navigators that will need to be developed based on nuances of the sample type. We have developed automated or semi-automated tools (part of our myOLARIS-toolbox) to report on Navigators which we will continue to expand to streamline clinical analysis. Using the flowchart described in Figure 7, we demonstrate how Navigators provide useful checkpoints to ensure quality sample processing and data acquisition for precision urine metabolomics. This is the first step towards biomarker discovery and is enabling clinical diagnostic development across a broad range of applications.

## Figures and Tables

**Table 1:** Sum-based Thresholding Minimizes False Positives.

Analysis	FP Noise-Based	FP Sum-Based
1.0054 vs. 1.0097	0	0
1.0054 vs. 1.0187	79	3
1.0054 vs. 1.0275	128	8
1.0097 vs. 1.0187	20	0
1.0097 vs. 1.0275	80	9
1.0187 vs. 1.0275	0	0

FP (false positives) are defined as features that were significantly different (FDR-*p*-value < 0.05 and FC > 1.5) using a KW test of significance.

**Table 2:** Impact of pH on False Positives.

Analysis	N. Features	Passing by Chance	Passing KW	Passing KW + FC	Passing FDR	Passing FDR + FC
pH 4.99 vs. pH 6.08	168	8.4	22	2	0	0
pH 4.99 vs. pH 8.08	167	8.35	36	6	0	0
pH 6.08 vs. pH 8.08	169	8.45	16	3	0	0

Listed are the number of features that pass a KW test of significance (KW), the KW and FC cut-off of >1.5, (KW + FC), FDR-adjusted *p*-value significance (FDR) and FDR and FC cut-off (FDR + FC) for each analysis.

**Table 3:** myOLARIS Navigators for NMR Precision Urine Metabolomics.

Navigator	Sources of Error	Description	Automatable?
1D ^1^H DSS	*Chemical shift differences*	Reference DSS peak to 0 ppm	Y
1D ^1^H DSS PW	*Protein contamination; poor shimming*	Indicator for lack of protein contamination and proper shimming if PW > 2 Hz	Y
1D Nav1:DSS AUC ratio	*Incomplete or inconsistent sample preparation*	Monitor sample processing quality with ratio < 0.8 flagging potential sample processing error(s)	Y
2D ^1^H-^13^C HSQC Nav1:creatinine	*Inconsistent sample preparation of disease confounder*	Ratio can aid in predicting SG of original sample	Y
1D ^1^H DFTMP	*Improper clustering across samples*	Determines sample pH and clusters pH-sensitive peaks	Y

Abbreviations: DSS: deuterated sodium 2,2-dimethyl-2-silapentane-5-sulfonate; Nav1: Navigator 1; PW: peak width; DFTMP: 1,1-difluoro-1-trimethylsilanyl methylphosphonic acid; HSQC: heteronuclear single quantum coherence spectroscopy.

## Data Availability

The raw data supporting the conclusions of this article will be made available by the authors on request.

## Supplementary Materials

The following supporting information can be downloaded at: https://www.mdpi.com/article/10.3390/metabo14050275/s1, Figure S1: Five-year publication growth rate for top areas of omics research; Figure S2: Reference spectra of Nav1 (2-chloropyrimidine-5-carboxylic acid); Figure S3: Nav1, but not DSS, is impacted by sample processing errors; Figure S4: ^1^H-^13^C HSQC spectra of highly dilute urine samples at sum-based cutoff; Figure S5: Nav1:Creatinine can predict original sample SG; Figure S6: Nav1 is stable in a solution stored at 4 °C ~30 days; Figure S7: Nav1 addition does not alter sample resonances; Figure S8: Chemical shift perturbation due to pH variation; Figure S9: DFTMP can guide clustering of pH sensitive peaks; Figure S10: Nav1 and DSS as Navigators for plasma precision metabolomics; Table S1: Pearson correlation coefficient of variables to manual cut off; Table S2: SG clean-up eliminates false positives; Table S3: Impact of sample preparation conditions on Nav1 and DSS ^1^H AUC.
