# Supplementary material for: NMR Precision Metabolomics: Dynamic Peak Sum Thresholding and Navigators for Highly Standardized and Reproducible Metabolite Profiling of Clinical Urine Samples
Source: Metabolites. 2024 May 10;14(5):275. doi: 10.3390/metabo14050275 (PMC11122845; doi:10.3390/metabo14050275)
Supplement: Supplementary file 1 [file metabolites-14-00275-s001.zip › metabolites-2981166-supplementary.pdf]

A

Year	2017	2018	2019	2020	2021	2022
metabolomics	5479	6401	7872	9512	11439	12790
proteomics	11663	12091	13000	14535	16221	15442
transcriptomics	19554	20927	24343	27407	30977	28979
genomics	85799	87876	94105	101790	110568	103556
epigenomics	2029	2262	2520	2904	3254	3017

B

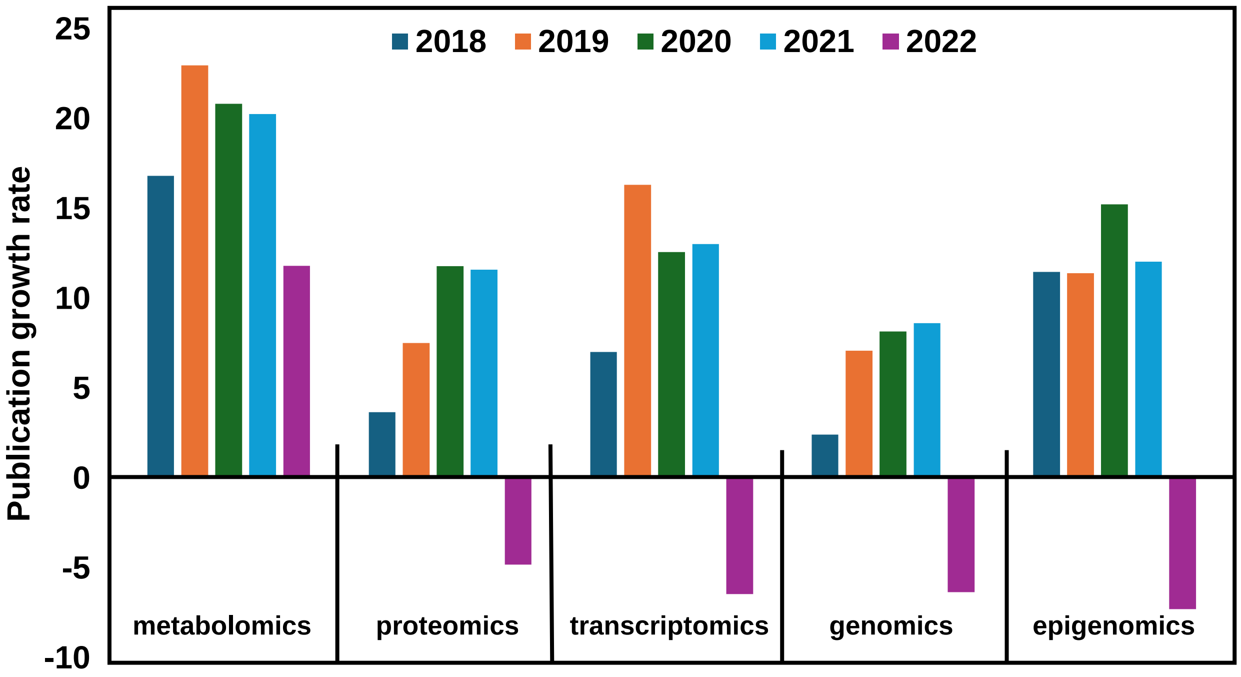

Figure S1. Five-year publication growth rate for top areas of omics research. (A) A PubMed search of number of articles published from 2017 to 2022 in metabolomics, proteomics, transcriptomics, genomics and epigenomics was tabulated. **(B)** The growth rate was calculated by determining the difference between two adjacent years, divided by the first year. For each year, the growth rate of metabolomics is greater than all other omic technologies during the last 5 years.

Table S1. Pearson correlation coefficient of variables to manual cut off.

Variables	Description	Correlation
manual_cutoff	Best intensity cutoff manually set by metabolite scientist	1.000
sum_gt30xnoise	Sum of intensity from peaks greater than 30 times the noise	0.994
sum_gt60xnoise	Sum of intensity from peaks greater than 60 times the noise	0.994
sum_pos	Sum of intensity from al positive peaks	0.994
sum_gt10xnoise	Sum of intensity from peaks greater than 10 times the noise	0.994
cutoff_top250	The cutoff which keeps the 250 peaks with highest intensity	0.993
creatinine_3.04x33.06	The intensity of creatinine peak at 3.04x33.06	0.993
alanine_1.49x19.00	The intensity of alanine peak at 1.49x19.00	0.990
creatinine_DSS_ratio	The intensity ratio of creatinine to DSS	0.984
peak_num_gt30xnoise	Number of peaks greater than 30 times the noise	0.984
peak_num_gt60xnoise	Number of peaks greater than 60 times the noise	0.982
glucose_5.26x94.86	The intensity of glucose peak at 5.26x94.86	0.976
sum_pos_dss_ratio	The ratio of sum of all positive peaks to DSS	0.973
peak_num_gt10xnoise	Number of peaks greater than 10 times the noise	0.973
peak_num_pos	Number of all positive peaks	0.973
mean_gt10xnoise	Average of intensity from peaks greater than 10 times the noise	0.958
mean_pos	Average of intensity from all positive peaks	0.958
alanine_1.42x19.32	The intensity of alanine peak at 1.42x19.32	0.954
median_pos	Median of intensity from all positive peaks	0.948
median_gt10xnoise	Median of intensity from peaks greater than 10 times the noise	0.948
mean_gt60xnoise	Average of intensity from peaks greater than 60 times the noise	0.948
mean_gt30xnoise	Average of intensity from peaks greater than 30 times the noise	0.939
Noise	Spectra noise from parameters.txt	0.927
peak_num_total	Number of all positive peaks	0.922
median_gt30xnoise	Median of intensity from peaks greater than 30 times the noise	0.860
median_gt60xnoise	Median of intensity from peaks greater than 60 times the noise	0.831
formate_8.47x14.00	The intensity of formate peak at 8.47x14.00	0.723
sum_pos_creatinine_ratio	The ratio of sum of all positive peaks to creatinine	-0.517
dss_intensity	Intensity of DSS	-0.680

Table S2. SG clean-up eliminates false positives

Analysis	N. of features	Passing by chance	KW	KW+FC	FDR	FDR + FC
SG = 1.0363 vs SG = 1.0093 (Concentrated and rediluted)	205	10.25	105	20	81	18
SG = 1.0363 vs SG = 1.0092	204	10.2	112	18	93	17
SG = 1.0093 (Concentrated and rediluted) vs SG = 1.0092	200	10	36	1	0	0

Listed are the number of features that pass a KW test of significance (KW), the KW and FC cut-off of >1.5, (KW+FC), FDR-adjusted p-value significance (FDR) and FDR and FC cut-off (FDR+FC) for each analysis.

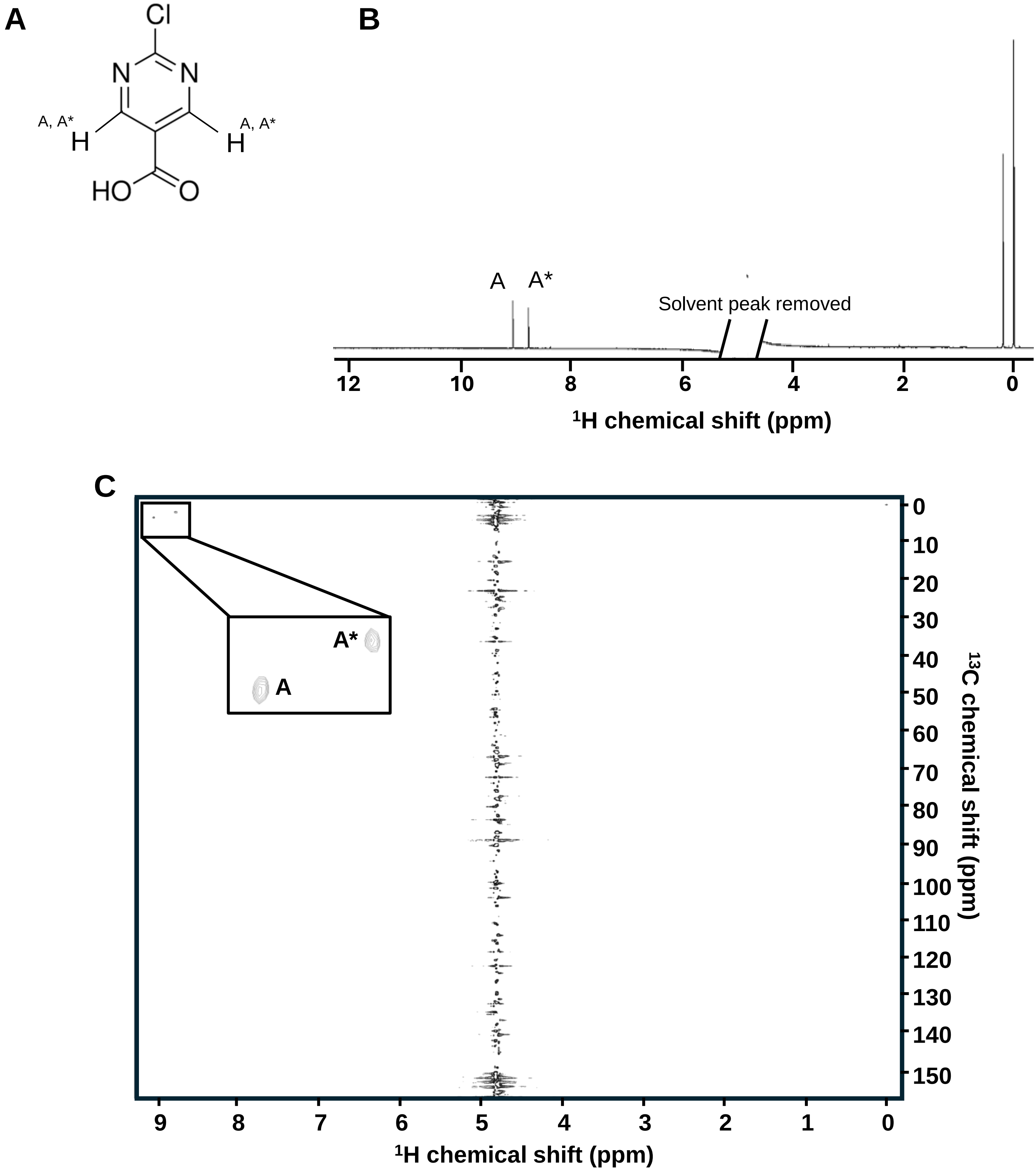

Figure S2. Reference spectra of Nav1 (2-chloropyrimidine-5-carboxylic acid). (A) Structure of 2-chloropyrimidine-5-carboxylic acid (CPC5A), used as Navigator 1 (Nav1). (B) ^{1}H and (C) ^{1}H-^{13}C HSQC reference spectra for Nav1. Signals A and A* are generated by the marked protons. Residual solvent signal is shown in the streak at 4.8 ppm.

Table S3. Impact of sample preparation conditions on Nav1 and DSS ^{1}H AUC.

Condition	Avg DSS AUC ±STD	Avg Nav1 AUC ±STD	Avg Nav1:DSS ±STD	%CV (Nav1 AUC)	%CV (Nav1:DSS)
Operator1 Day1	3.18E+12±4.79E+10	3.19E+11±4.16E+10	0.1±0.005	6.37	5.66
Operator2 Day1	3.16E+12±2.55E+10	3.05E+11±2.40E+10	0.096±0.007	7.86	7.98
Operator1 Day2	3.19E+12±4.22E+10	3.76E+11±1.22E+10	0.118±0.003	3.25	2.91
Operator2 Day2	3.21E+12±5.24E+10	3.70E+11±1.85E+10	0.115±0.006	5.82	6.66
Operator1 Day1-Day2	3.18E+12±4.19E+10	3.51E+11±3.36E+10	0.11±0.01	9.65	9.27
Operator2 Day1-Day2	3.18E+12±4.68E+10	3.38E+11±3.97E+10	0.105±0.011	11.77	11.31
Operator1+2 Day1-Day2	3.10E+12±3.81E+11	3.42E+11±3.61E+10	0.107±0.01	10.55	10.16
Skipping CHCl3	3.15E+12±4.76E+10	3.07E+11±8.76E+09	0.097±0.002	NA	NA
Skipping water	3.21E+12±6.88E+10	2.43E+11±3.87E+10	0.075±0.012	NA	NA
Losing 1/3 Aqueous phase	3.24E+12±4.56E+10	2.20E+11±1.07E+10	0.067±0.003	NA	NA
Losing 1/2 Aqueous phase	3.01E+12±5.52E+11	1.69E+11±3.02E+10	0.056±0.004	NA	NA

Listed are the average (avg) AUC and standard deviation (STD) for DSS, Nav1 and Nav1:DSS AUC ratio in ^{1}H spectra. The coefficient of variation (%CV) for Nav1 and Nav1:DSS ratio is provided for each condition where applicable.

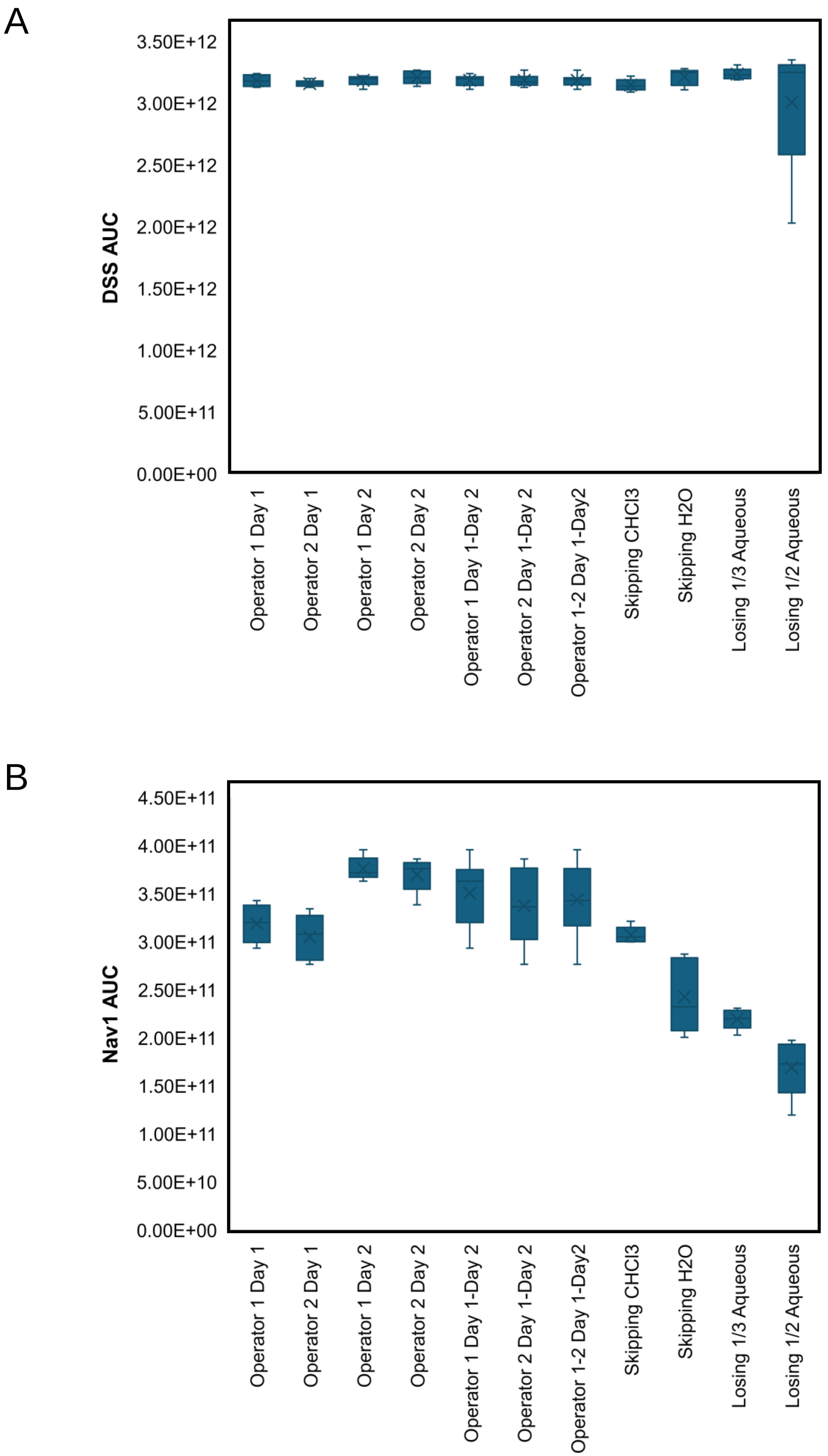

Figure S3. Nav1, but not DSS, is impacted by sample processing errors. (A) The 1H AUC of DSS is consistent for nearly all conditions while, the 1H AUC of Nav1 (B) significantly varies based on variations in sample processing.

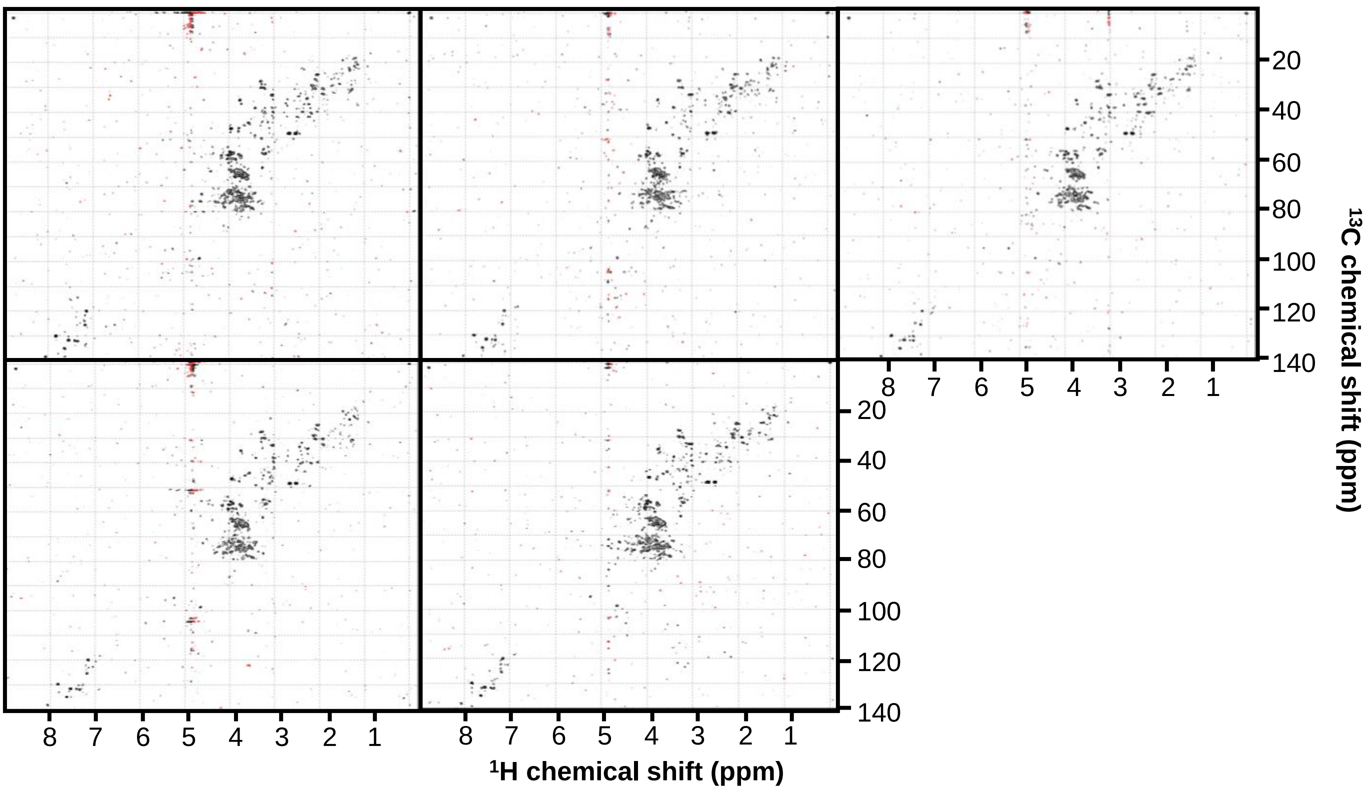

Figure S4. ^{1}H-^{13}C HSQC spectra of highly dilute urine samples at sum-based cutoff. Five replicates of urine at a dilute SG of 1.0012 show high levels of noise that will add unwanted intensity signals and thus should not be used for analysis.

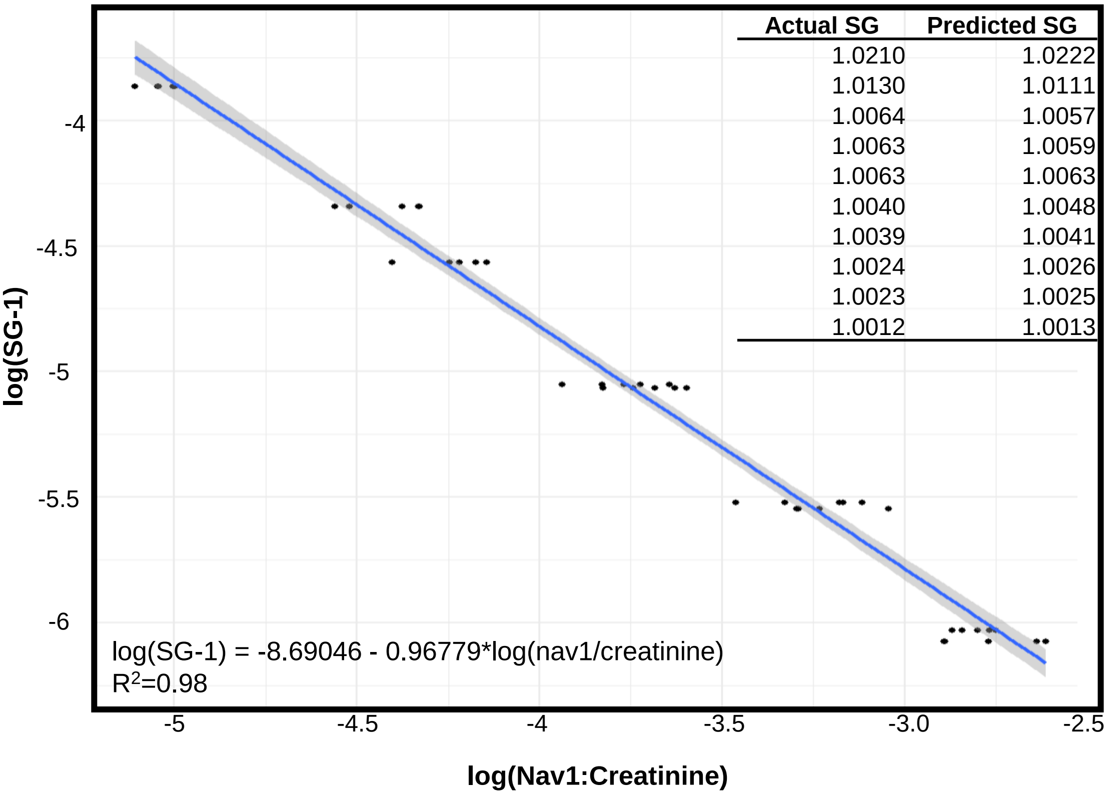

Figure S5. Nav1:Creatinine can predict original sample SG. A model was built using the equation reported in the figure to calculate original sample SG from Nav1:Creatinine. A strong correlation between these two variables is demonstrated (R^2=0.98). The 95% CI range is shown in light blue.

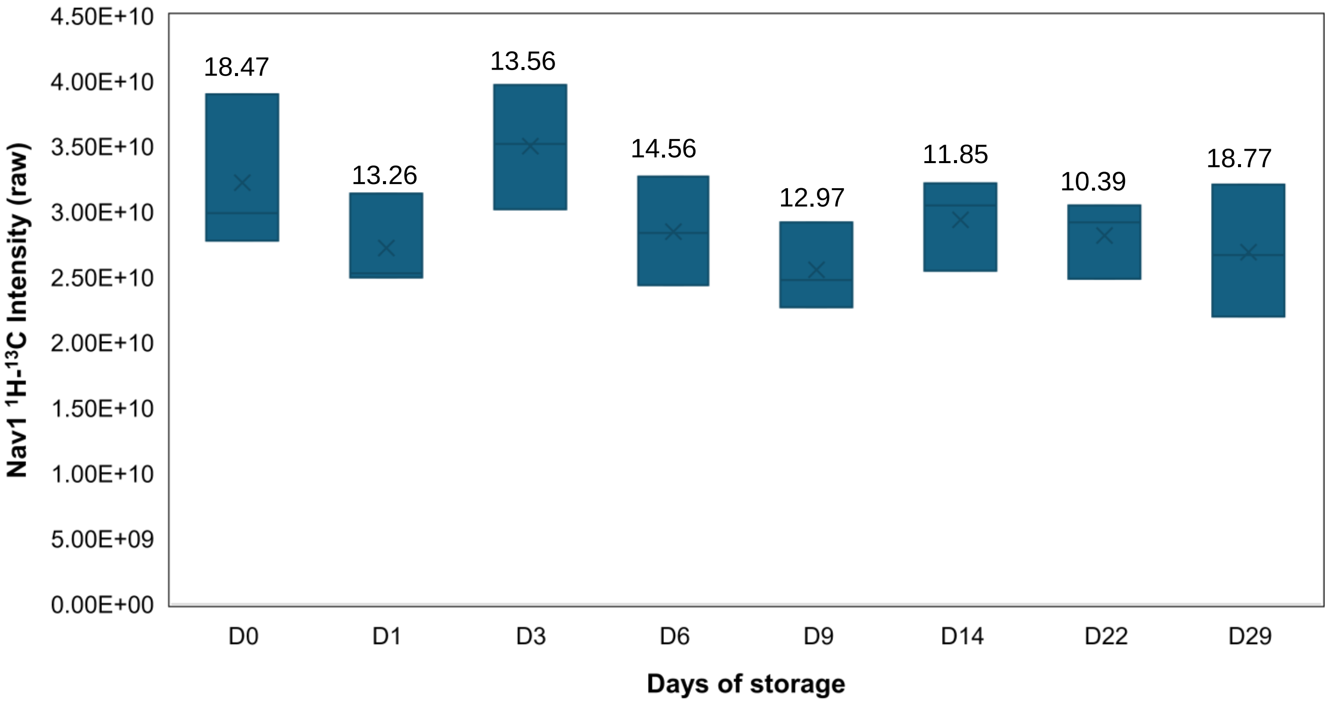

Figure S6. Nav1 is stable in a solution stored at 4 °C ~30 days. Nav1 ^{1}H-^{13}C intensity shows minimal variation at 4 °C up to 29 days. %CV for each day is reported on top of the box plots and the total percent CV is 16% across 29 days.

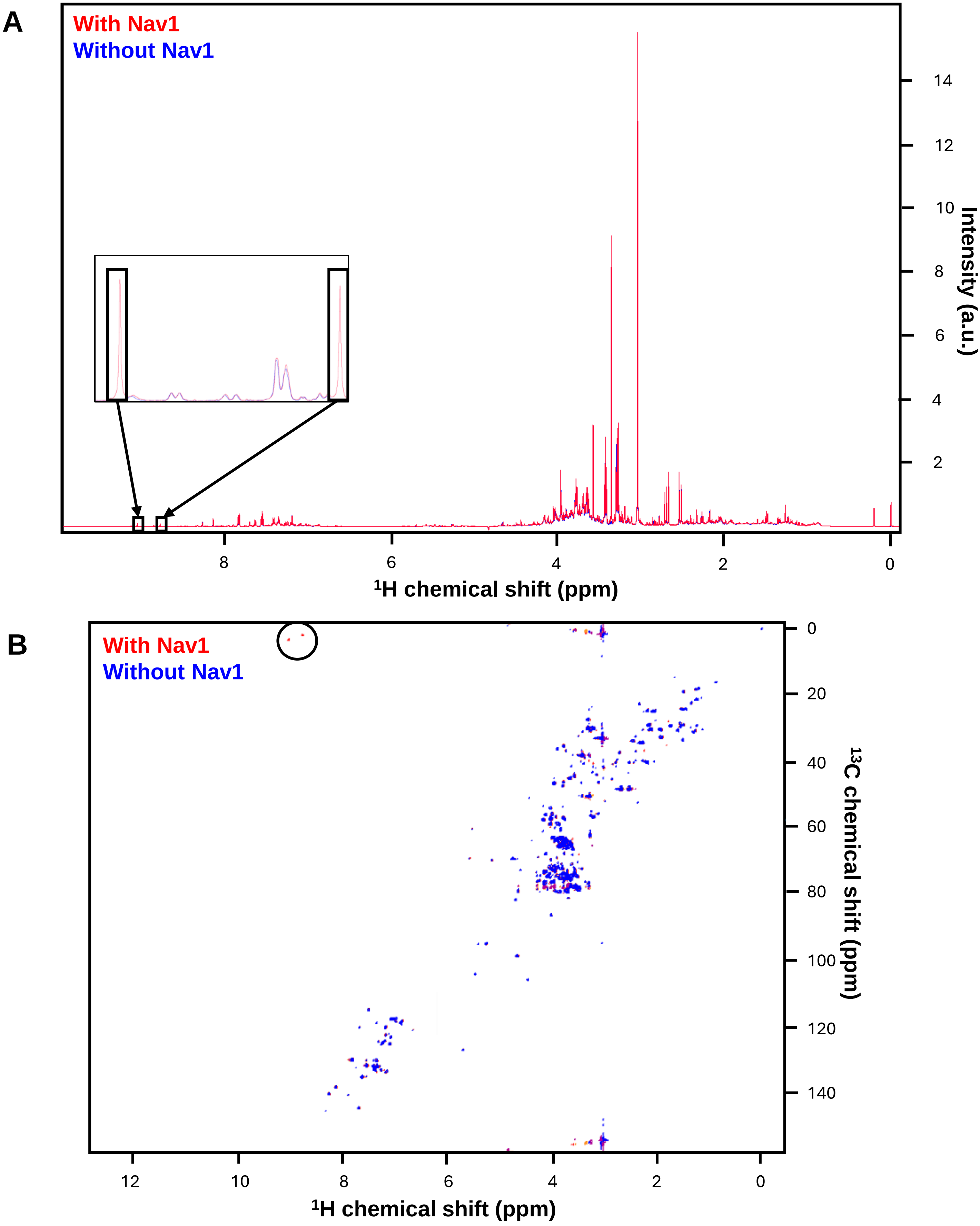

Figure S7. Nav1 addition does not alter sample resonances. The addition of Nav1 does not alter metabolite resonances with little to no spectra differences observed in (A) the 1D and (B) the 2D with (red) and without (blue) the addition of Nav1 at the beginning of sample processing. A zoom-in of the region containing Nav1 peaks is shown in A.

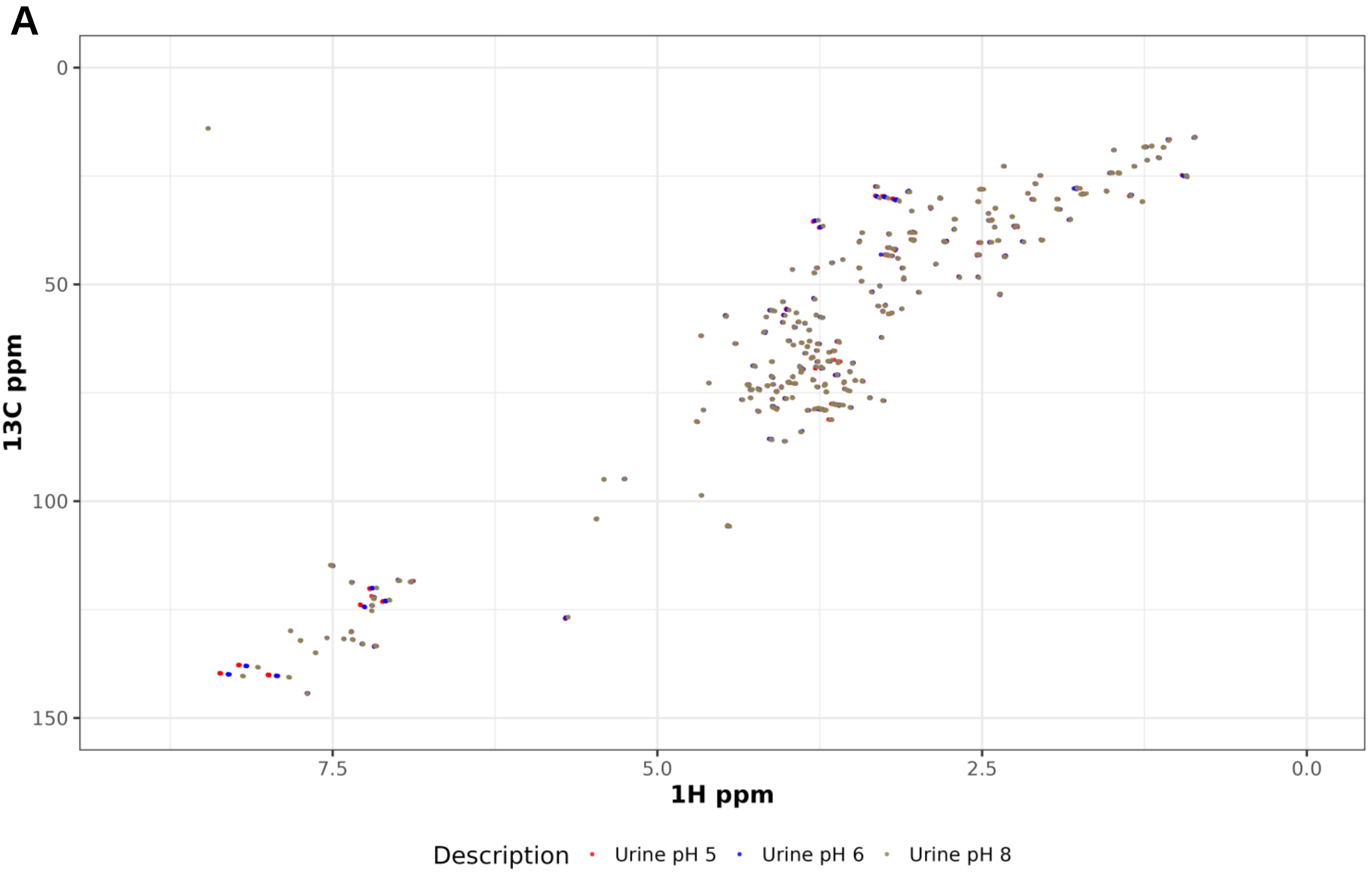

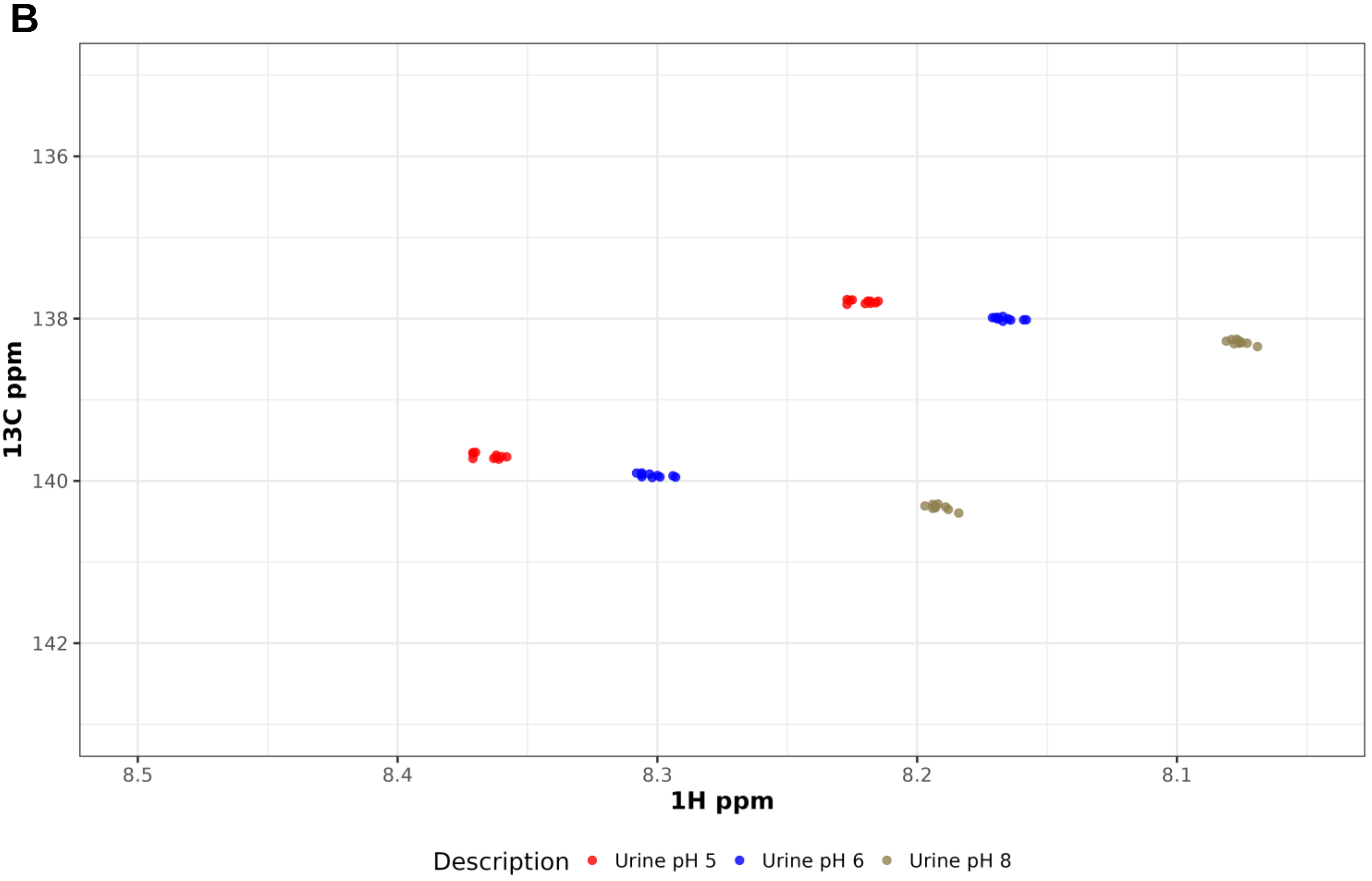

Figure S8. Chemical shift perturbation due to pH variation. (A) Overlay of resonances from 30 urine spectra samples with initial pH of 5 (red), 6 (blue), and 8 (brown), displaying pH-dependent chemical shift perturbations. (B) Zoomed in the region of 8-8.5x135-143 in ^{1}Hx^{13}C ppm highlight significant chemical perturbations across samples.

A

Sample	DFTMP hppm	Initial pH	Final pH
1	0.20392	4.99	6.8
2	0.20338	4.99	6.82
3	0.20374	4.99	6.8
4	0.2036	4.99	6.82
5	0.20338	4.99	6.81
6	0.20227	6.08	6.94
7	0.20245	6.08	6.94
8	0.20254	6.08	6.93
9	0.20217	6.08	6.94
10	0.20248	6.08	6.94
11	0.20066	8.08	7.14
12	0.20112	8.08	7.14
13	0.20063	8.08	7.14
14	0.20082	8.08	7.14
15	0.20066	8.08	7.14
16	0.2039	4.99	6.79
17	0.20384	4.99	6.79
18	0.20375	4.99	6.79
19	0.20344	4.99	6.81
20	0.20341	4.99	6.81
21	0.20199	6.08	6.95
22	0.20245	6.08	6.93
23	0.20239	6.08	6.95
24	0.20245	6.08	6.93
25	0.20217	6.08	6.94
26	0.20088	8.08	7.14
27	0.20094	8.08	7.15
28	0.20109	8.08	7.14
29	0.20091	8.08	7.14
30	0.20109	8.08	7.13

B Final pH = -119.7*DFTMP + 31.174

Initial pH = -1096.4*DFTMP + 228.17

C

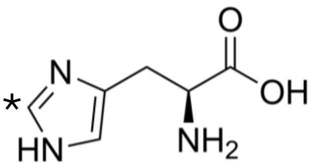

Predicted Initial pH = 4.53
Predicted Final pH = 6.79

D

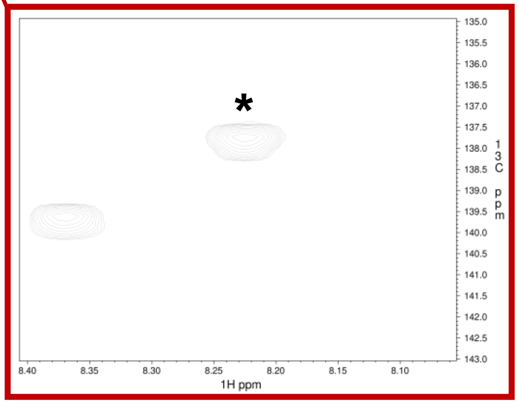

Predicted Initial pH = 6.37
Predicted Final pH = 6.98

E

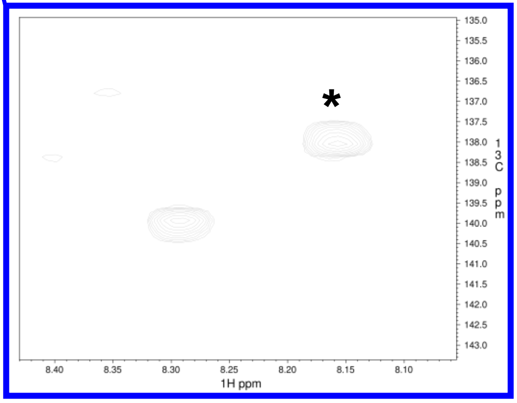

Predicted Initial pH = 8.16
Predicted Final pH = 7.17

F

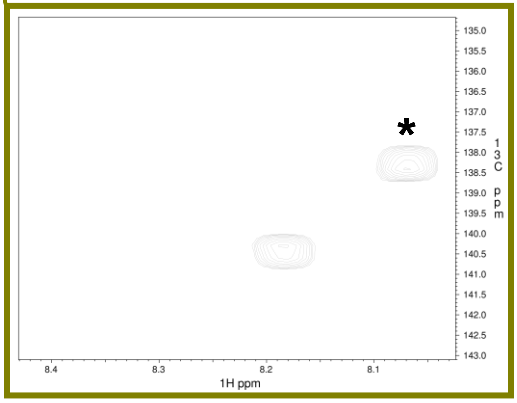

Figure S9. DFTMP can guide clustering of pH sensitive peaks. (A) In a cohort of 30 samples with different initial and final pH, the chemical shift for DFTMP in 1D 1H NMR spectra was measured. (B) Equations to predict initial and final pH by using DFTMP chemical shift. (C) Structure of histidine highlighting (*) one of the pH-sensitive protons. (D-F) Examples of the pH-sensitive histidine peak in 1H-13C HSQC urine spectra with different pH. Knowing the pH of each sample empowered all histidine peaks which had a chemical shift variation of 0.17 in both ^{1}H and ^{13}C across the cohort to be clustered as a single feature.

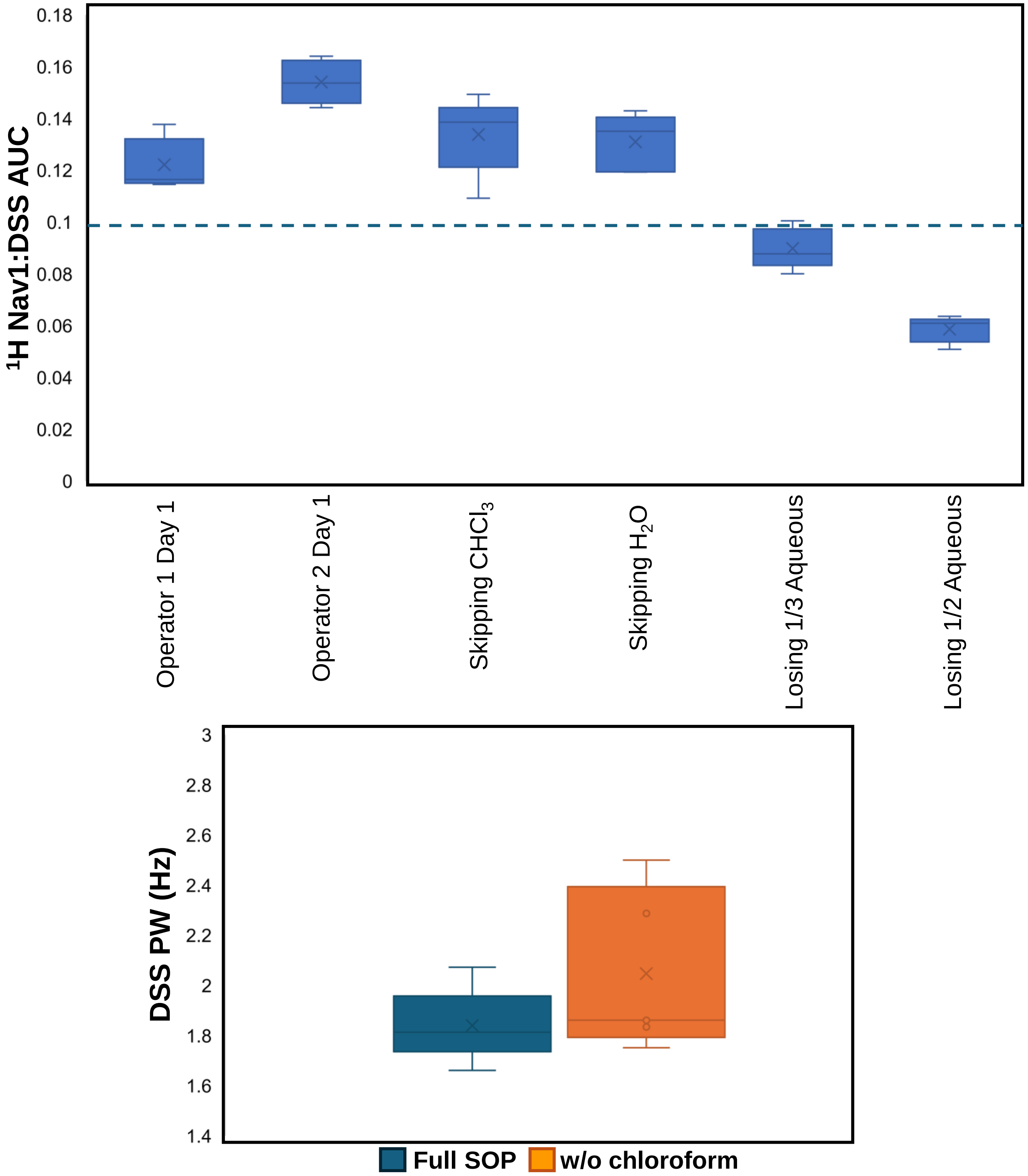

Figure S10. Nav1 and DSS as Navigators for plasma precision metabolomics. (A) ^1H Nav1:DSS AUC ratio is a sensitive indicator for variations in sample processing, wherein a ratio of < 0.1 (dotted line) signals suboptimal metabolite extraction. **(B)** In metabolites extracted from plasma, DSS PW at half-height is ≤ 2 Hz following the full SOP. Samples in which protein precipitation steps are skipped (i.e., without the chloroform addition) have a greater range and higher average peak width value. Five replicates for each condition were tested with standard deviation represented by error bars.
